# Silicon Nanowire-Based Devices for Gas-Phase Sensing

**DOI:** 10.3390/s140100245

**Published:** 2013-12-24

**Authors:** Anping Cao, Ernst J.R. Sudhölter, Louis C.P.M. de Smet

**Affiliations:** Department of Chemical Engineering, Delft University of Technology, Julianalaan 136, Delft 2628 BL, The Netherlands; E-Mails: a.cao-1@tudelft.nl (A.C.); e.j.r.sudholter@tudelft.nl (E.J.R.S.)

**Keywords:** silicon nanowire, field effect transistor, resistor, gas, vapor, volatile organic compound

## Abstract

Since their introduction in 2001, SiNW-based sensor devices have attracted considerable interest as a general platform for ultra-sensitive, electrical detection of biological and chemical species. Most studies focus on detecting, sensing and monitoring analytes in aqueous solution, but the number of studies on sensing gases and vapors using SiNW-based devices is increasing. This review gives an overview of selected research papers related to the application of electrical SiNW-based devices in the gas phase that have been reported over the past 10 years. Special attention is given to surface modification strategies and the sensing principles involved. In addition, future steps and technological challenges in this field are addressed.

## 1. Introduction

Rapid ongoing industrial developments and further quality of life improvements put a large demand on the sensitive and selective detection of molecules in the gas phase for environmental monitoring, process control and safety, and medical diagnostics purposes [1]. Gas sensors were first mainly used in coal mines where online and precise monitoring of hazardous gases has to be carried out continually, in order to assure work place safety [2]. Soon after, gas/vapor sensors also began to appear in the chemical industry, environmental pollution monitoring and the human health fields, for instance, in the detection of explosive gases in the hydrogen production industry and methane distribution networks, air-quality monitoring in urban areas, breath analysis for traffic safety and non-invasive medical diagnostics [3].

Various nanomaterials and nanostructures present a promising basis for high-performance sensing devices [4], such as sensors based on nanoparticles (NPs) [5], nanotubes (NTs) [6] or nanowires (NWs) [7]. A key property of these nanostructured materials is their high surface-to-volume ratio, and also that one or more of the physical dimensions are less than or comparable to the charge screening length, *i.e.*, the Debye length. Therefore, these nanostructured materials very often exhibit superior sensitivity in chemical surface processes [8].

Among all these nanomaterials, silicon nanowires (SiNWs) are very good candidates for sensing applications due to several advantages they present. For example, electrical devices made from SiNWs allow one to analyze responses not only by the voltage between the electrodes, but also by a gate voltage [9]. Also, they have a relatively large carrier mobility [10] and are tunable by controlling the doping level [11]. Compared to devices prepared from carbon nanotubes and organic materials like wires from conducting polymers, SiNW-based devices are more compatible with very-large-scale integration (VLSI) processes and complementary metal–oxide–semiconductor (CMOS) technologies [12,13]. In addition, in terms of the fundamental sensor mechanism, gas sensors based on SiNWs are better understood than devices based on metal-oxide nanowires and polymer nanowires [14]. Finally, the ability to chemically modify the surface of SiNWs enables not only the chemical immobilization of selector materials, but it also affects the device performance [15,16]. Recently we reviewed the different surface modification strategies that have been explored to modify SiNW-based devices [17].

The field of SiNW (sensor) devices was opened up by the lab of Lieber who reported on the fabrication [18] of SiNWs-based sensors and their use in the detection of chemical and biological species [19]. In their novel approach, the functionalization of SiNWs with oxide/amines, biotin, antigens, or the calcium-binding protein calmodulin allowed real-time detection of protons, streptavidin, antibodies, and calcium ions, respectively, and all the detections were reported to exhibit a high and specific sensitivity. Since the pioneering work of Lieber, SiNWs have been widely studied as sensors by many researchers due to their capabilities in sensitive, label-free and real-time detection of biological and chemical species, coupled to their uniformity and reproducibility, as well as excellent scalability and manufacturability for mass production by relatively simple preparation methods, benefitting from mature fabrication technologies [20].

A search using the keywords “silicon nanowire” and “sensor” within the Web of Science yields more than 600 papers over the past 12 years. Most of these studies are on the detection of analytes (target compounds) in aqueous environments, mainly within the context of biosensing. However, only several dozens of studies address sensing in the gas phase. Hence, in order to obtain a deep understanding of the gas-sensing mechanism in (modified) SiNW-based devices and to show and discuss the diverse approaches in device fabrication, this work aims to give an overview of most of the research papers related to the sensing in gas phase using electrical devices that consist of both in-plane orientated and vertical-standing SiNWs. For reviews on fabrication methods of SiNWs, we recommend the following contributions: several with a clear focus on the chemical-vapor-deposition fabrication of NWs by the Lieber group [21–23] or one on top-down fabricated SiNW-based sensors from the Reed group [24]. For an extensive and very recent overview of different nanomaterials that have been explored to prepare NW-based gas sensors and volatile organic compound (VOC) sensors, we refer to the review by Chen *et al.* [25] and the recent reviews by Haick's group [26,27]. Finally, last year Penner wrote a review on chemical sensing with nanowires, that includes relevant sections on SiNW-based devices and also on examples of gas sensing, mostly associated with polymers and metal oxides [14].

In the present review, first the most common SiNW device configurations and the general sensing mechanisms are described in Section 2. Then, a summary of the device specifications, including the modification methods applied, target compounds involved and the sensing performance of different SiNW-based devices is presented in Table 1. The reviewed work has been divided in three different categories: gas sensors, vapor sensors, volatile organic compound sensors. In Section 3, selected contributions from these three categories are reviewed and discussed in more detail. In all cases, we briefly start with the relevance of measuring the target compounds. Focus is given to the different surface modification strategies that are applied onto mostly surface-oxidized SiNWs, and various signal enhancement methods by detecting the change of electrical properties. In Section 4 we conclude the review and present an outlook.

## 2. Working Principle: Resistor *vs.* Field Effect Transistor

There are different ways of classifying devices associated with SiNWs. The first one is related to the fabrication process of the nanowires. Nanowires can be grown from precursors, individual atoms and molecules to build the desired nanostructures, in some cases through smart use of self-assembly [21]. These are referred to as bottom-up fabrication methods and typically require the transfer and deposition of the nanowires onto a substrate, followed by the fabrication of contact pads. Alternatively, one can make use of top-down methods starting with patterns made on a large scale and then reducing the lateral dimensions to the nanoscale [24]. The first paper on SiNW-based sensor devices made use of bottom-up approaches [18], but an increasing number of studies deal with top-down methods, mainly because they have the advantage of reproducibility and reliability, improved contact properties and the (high density) integration possibilities related to CMOS processes. A comparison between the two fabrication approaches is the object of other reviews [28,29].

Another approach on categorizing SiNW-based sensors is based on way the electrical characterization or read-out is performed. In cases where changes in the nanowire resistance (*i.e.*, current) are measured without the use of a so-called front-gate or back-gate electrode, one typically refers to resistor-based sensors. On the other hand, devices that make use of an applied gate are referred to as Field Effect Transistors (FETs). It is noted that there is no intrinsic relationship between the type of electrical read-out (resistor *vs.* FET) and the way of preparing the SiNWs (grown or top-down made). At the same time it is noticed that these days most NW FETs are prepared via top-down approaches.

**Figure 1.**
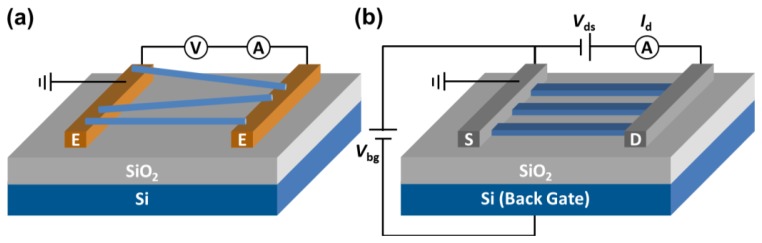
Simplified schematics of the SiNW-based (**a**) resistor and (**b**) SiNW-based FET to illustrate the differences in the electrical configuration and the way the nanowires are orientated with respect to the electrodes (E) and the source (S) and drain (D).

Figure 1a depicts the schematics of the resistor-based configuration. Here, the SiNWs bridge the positive and negative electrodes to allow a current flow. The adsorption of the analyte onto the SiNW surface alters the surface state. By monitoring the resulting change of electrical resistance or conductance using a simple direct current (DC) circuit the analyte can be detected [25]. In the case of the FET-based configuration, the SiNW functions as a conductive channel. The terminal ends are now connected by so-called source and drain contacts, typically metallic or highly-doped semiconductor materials. The number of charge carriers and thus the conductance through the SiNW can be changed by a third gate electrode, either via a back gate (as depicted in Figure 1b) or a front gate. For example, by applying a back-gate potential (*V*_bg_) the nanowire can be brought into the depletion-mode, enabling one to measure in the subthreshold regime where the transducer is most sensitive [9]. Any (bio)chemical or recognition event that occurs near the nanowire surface influences the local electrical voltage experienced by the nanowire and may change the extent of depletion. The number of majority charge carriers varies, which is registered as a change in drain current (*I*_d_) if a fixed source-drain voltage (*V*_ds_) is applied. Another way of registering the recognition event is by adaption of the back-gate voltage Δ*V*_bg_ in such a way that at fixed *V*_ds_, *I*_d_ is kept constant. In that case Δ*V*_bg_ reflects the change in boundary potential at the interface of the nanowire and its environment. Due to the large surface-to-volume ratio of nanowires and the gate effect of the amplifier configuration, nanowire-based devices have high potential for the development of single-molecule detection [30].

## 3. Gas-Phase Sensing

Table 1 gives an overview of selected electrical sensor devices associated with SiNWs that have been studied in the gas environment. It is noted that a gas is a substance that has a single defined thermodynamic state at the temperature of investigation, while a vapor is a substance in the gas phase at a temperature lower than its critical point. So according to this definition, VOCs are vapors as well, but most literature on sensing in a gas phase makes use of the following sub-classification: inorganic (gases and vapors) and organic (explosives, nerve agents and VOCs) compounds. Within one category the studies are listed in order of their appearance in Table 1. Most, but certainly not all examples, cover devices with in-plane orientated SiNWs. With top down and bottom up (*i.e.*, nanowire growth) we refer to the approach of SiNW fabrication, although in some cases top-down prepared SiNWs are transferred to another surface. It is noted that here we are interested in the electrical properties of the nanowires and hence the length is defined by the spacing between the two electrodes in those cases where SiNWs have been placed onto electrodes (while the actual length of these nanowires could be longer). Nanowires that have not been functionalized are referred to as “bare”, *i.e.*, a thin oxide layer. As most studies aim to study the detection limits, data on the sensor sensitivity (s) and the measured concentration (mc) range are listed as well.

### 3.1. Inorganic Gases

#### 3.1.1. Nitrogen Dioxide (NO_2_)

**Figure 2.**
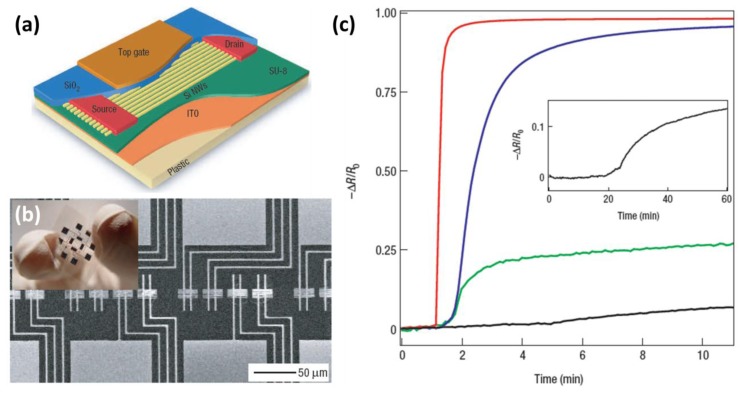
Electrical characterization of nanowire thin-film transistors on plastic. (**a**) Schematic illustration of the active area of a transistor, with the electrodes and various layers labeled; (**b**) Scanning Electron Microscope image of the sensor platform. Each device (horizontal strip) is contacted by two Ti electrodes (oriented vertically) that extend to larger pads (top and bottom image edges). Inset: Digital photograph of the flexible sensor chip; (**c**) Electrical response of a nanowire-based sensor to 20 ppm (red curve), 2 ppm (blue curve), 200 ppb (green curve) and 20 ppb (black curve) NO_2_ diluted in N_2_. The gas is introduced to the sensing chamber after 1 min of flowing N_2_. Inset: An extended response of the sensor to 20 ppb NO_2_; the gas is introduced after 20 min of flowing N_2_. This figure is composed of figures taken from [31], reprinted with permission from the Nature Publishing Group ©.

The ability to accurately monitor NO_2_ concentrations in air is very important, because NO_2_ is a potentially toxic gas that can lead to respiratory symptoms in humans and detrimentally influence the growth of plants [67]. In addition, NO_2_ can lead to the formation of ground-level smog and acid rain. The atmospheric concentration of NO_2_ is typically around 0.01 ppm, while values above 0.65 ppm are very unhealthy as reported by the U.S. Environmental Protection Agency [68]. In 2007, Heath and co-workers reported a study on a NO_2_ sensor based on SiNWs, made by transferring hundreds of pre-aligned, top-down prepared SiNWs from a silicon-on-oxide (SOI) wafer onto plastic [31]. Layers of deposited Ti and SiO_2_ were used to fabricate source/drain contacts and a gate, respectively. Figure 2a presents a schematic illustration of the active area of thin-film transistors (TFTs) made by this so-called superlattice nanowire pattern transfer (SNAP) approach, while Figure 2b shows a Scanning Electron Microscope image of the sensor platform and a photograph of the flexible sensor chip.

The changes in the nanowire resistance were measured and further analyzed when sensor chips were exposed to NO_2_ and also a series of VOCs in nitrogen. Figure 2c shows the normalized response of a SiNW sensing element to different NO_2_ concentrations, clearly showing that NO_2_ concentrations down to at least 20 ppb can be detected. It was concluded that due to the strong electron-withdrawing capabilities of NO_2_, the withdrawing of electrons from the p-type Si causes hole accumulation and thus an increase in the conductance when it is exposed to NO_2_ in nitrogen. In these experiments the nanowires had a 25 nm thick SiO_2_ layer as a gate dielectric. The SiO_2_ surface was also modified using silanization to obtain aldehyde-, alkane- and amino-terminated layers. This way a “nano-electronic nose” library was built, and the devices were found to be capable to distinguish low concentrations of acetone and hexane vapors via an analytical mapping of the array response patterns. The authors report that the mechanism of these responses may result simply from vapor-wire dipole-dipole effects, but could also involve dehydration of the surface, displacement of adsorbed oxygen and/or changes in surface-charge screening. To conclude, this interesting combination of highly sensitive SiNWs and flexible plastic support could open up opportunities in portable, wearable or even implantable sensors.

Soon after, a series of three studies from the Fudan University and the Rutherford Appleton Laboratory was published on different fabrication strategies of p-type SiNW-based sensors for the detection of NO_2_. All studies are based on a bilayer (SU8/PMMA) or trilayer (SU8/SiO_2_/PMMA) nanoimprint lithography method, which was combined with different process steps, including reactive ion etching (RIE) [33], or wet etching [35], and angle deposition [38]. In the case of the wet-etching approach the cross-section of the SiNWs was trapezoidal, while in the other cases rectangular cross sections were obtained. In all cases the silicon nanowires are covered with SiO_2_ and a photolithography step was taken to define the Al contact pads. The resulting devices have been analyzed as a resistor (so no gate). In more detail, changes in the current have been recorded upon changing the environment of only N_2_ to 250 ppm NO_2_ in N_2_. It is noted that only this concentration was studied and that—in contrast to McAlpine *et al.*—the system was not tested for lower NO_2_ concentrations. The detection mechanism was explained along the lines of the study of the Heath group: the exposure to NO_2_ lead to an increase in the hole concentration, in the region near the surface of the p-type SiNW due to its electron withdrawing properties. In all three studies, two different SiNW widths were compared and in all cases a higher sensitivity was observed for NWs with the smaller width.

**Figure 3.**
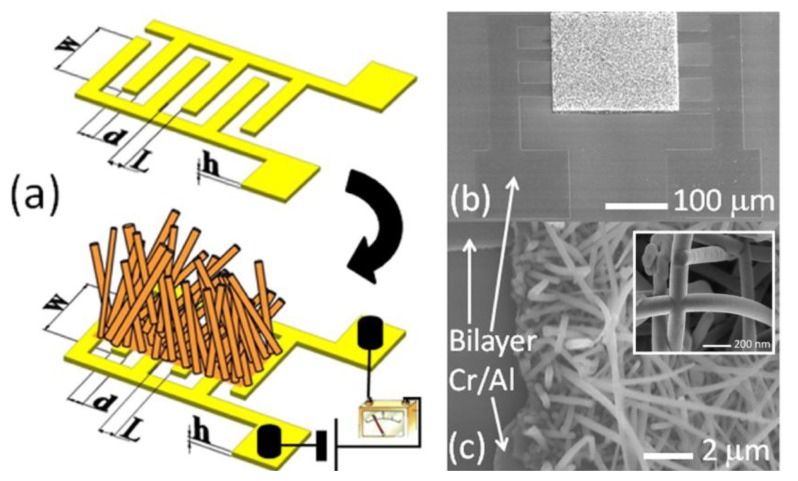
(**a**) A schematic representation of the interdigitated structure used as a substrate to grow SiNWs. *W* = 200 μm, *L* = 3–21 μm, *h* =100 nm, SEM images of (**b**) the final chemoresistive sensor and (**c**) SiNW network detail onto the interdigitated structure; (**c**) The inset shows a SEM image of self-welding NW-NW junctions present in the SiNW network. This figure is composed of figures taken from [39], reprinted with permission from the American Institute of Physics ©.

In 2012, Cuscunà *et al.* reported an alternative on-chip fabrication method for devices with SiNWs, and tested the detection of NO_2_ [39]. While most bottom-up approaches for the SiNW-based sensors usually need the processes of removing and transferring the SiNWs from the growth substrate to another support, the authors exploited SiNWs directly grown onto a selected area, over and between pre-patterned, interdigitated Cr/Al electrodes defined on oxidized silicon wafers (Figure 3).

Subsequently, the SiNWs were modified with amino groups by plasma polymerization after removal of native oxide by wet etching. It was shown that the introduction of amino functionalities highly enhanced the capability to detect NO_2_, and even more in a humid environment (50% RH). This was rationalized by the authors as follows. Amines have a basic nitrogen with a lone pair capable to bind to strong electron-withdrawing molecules like NO_2_. An electron transfer from the modified surface to NO_2_ is established, causing changes in the electrical conductivity. The authors believe that NO_2_ can react with water, giving nitric acid (HNO_3_), which provokes the strong interaction with the amino-terminated SiNWs. What is not considered in this paper, however, is that the removal of the native oxide may also affect the device sensitivity as shown by Bunimovich *et al.* [16]. Furthermore it is stressed that this example makes use of a SiNW network, rather than single SiNWs. The fabricated device had a close connection between the SiNWs and electrodes and a large number of NW-NW self-welded junctions (Figure 3c, inset), which provided a very high conductance, resulting in a detection limit down to a few ppb. Based on these results, the authors concluded that the extension to other gases is possible by exploiting the existing knowledge on chemical modifications of the Si surface.

#### 3.1.2. Nitrogen Oxide (NO)

The detection of NO is important, because changes in NO levels can be indicative of certain illnesses such as Alzheimer's or asthma [69]. Currently, chemiluminescence is commonly used for the detection of NO gas in a patient's breath [70]. While this technique is extraordinarily sensitive, with a detection limit of 300 ppt, the required instrumentation is large, expensive, and needs various supporting accessories, such as vacuum pumps and an ozone generator. Accordingly, there is a need to develop new, smaller NO gas sensors with a sturdier, scalable production at low cost.

Lee and co-workers reported a NO sensor based on porous n-type SiNWs prepared by a metal-assisted chemical etching method [34]. NO was detected in dry, synthetic air. NO, here not oxidized to NO_2_ by O_2_ due to low concentrations, was adsorbed by high-density, vertically aligned porous SiNWs. Due to the strong electron-donating power of NO a charge transfer from NO to the SiNWs takes places, increasing the amount of electron carriers in and the conductance of the SiNWs. The sensor shows a fast response and excellent reversibility to sub-ppm NO concentrations. The interference of other gases has also been investigated and the as-prepared devices showed a small resistance for NH_3_ (at 3,000 ppm) and little resistance changes upon the exposure to benzene, methanol, ethanol and other (non-specified) organic vapors of which data are presented in the paper. While the mechanism is not discussed, NO seems to be the smallest molecule tested, so possibly a size-exclusion effect in the porous structure may explain the selectivity.

#### 3.1.3. Hydrogen (H_2_)

Hydrogen is used in many industrial processes such as hydrogenation, petroleum transformation, cryogenic cooling, and the chemical production of a variety of materials. As H_2_ is odorless, colorless, and flammable at concentrations over 4%, it poses safety concerns and creates a need for effective H_2_ leakage sensors with a lower limit of detection (LOD) to identify small leaks. A review on electrochemical H_2_ sensors by Korotcenkov *et al.* [71] also suggests the need for cost effective, low power, and compact sensors with a long-term stability, minimal cross-sensitivity and fast response.

Many H_2_ sensors reported to date are based on the selective absorption of H_2_ by palladium, which results in the reversible formation of palladium hydride (PdH_x_), changing the electrical and optical properties of Pd. Bare SiNWs do not show appreciable sensitivity to H_2_, so efforts have been made to find suitable functionalization schemes to decorate SiNWs with H_2_-sensitive materials. The Lee group built a Pd-functionalized SiNW-based sensor for H_2_ detection [32]. SiNWs were grown and their oxide layer was removed by immersion in hydrofluoric acid, then dipped into a saturated palladium chloride (PdCl_2_) solution to form a coating of Pd nanoparticles. The most applied method to reduce Pd^2+^ ions is by the addition of a reducing agent, e.g., ascorbic acid. Here the authors conclude that surface groups (*i.e.*, Si–H) act as a reductor. The modified SiNWs were subsequently dispersed onto a silicon wafer with a 300 nm oxide layer. Gold electrodes were deposited and *I*–*V* measurements were carried out with a two-probe analysis system. Upon exposure to 5% hydrogen, the current signal of the sensor increased about 20 times. The response time was three seconds only, which is much faster than that of the macroscopic Pd wire sensor. The sensing mechanism was explained by the Fermi level modulation upon chemical absorption of H_2_ in Pd nanoparticles that leads to the band diagram change at the metal-semiconductor interfaces.

**Figure 4.**
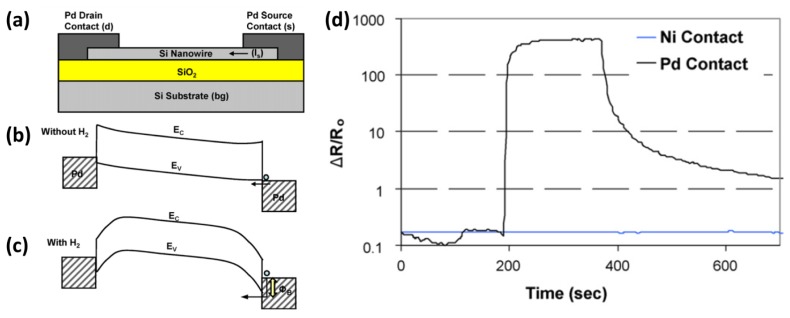
(**a**) Schematic of the SBNWFET, consisting of p-type doped nanowires; (**b**) Energy band diagram before H_2_ is introduced, where *E*_C_ and *E*_V_ stand for the energy levels of the conduction and valence band, respectively. The holes do not experience a barrier and the contact is Ohmic. The native SiO_2_ layer is omitted in this diagram because it is too thin to affect transport; (**c**) Energy band diagram after H_2_ is introduced, showing the formation of a Schottky barrier; (**d**) Comparison of the responses of Ni and Pd contacted NWFETs to 1% H_2_. This figure is composed of figures taken from [36], reprinted with permission from Elsevier B.V. ©.

To combine the benefits of SiNW-based sensors with the high performance reported for Schottky-based bulk sensors, Skucha *et al.* worked on the design, fabrication, and characterization of a H_2_ sensor based on a Pd/SiNW Schottky barrier field-effect transistor (SBNWFET) [36]. To form an array, grown SiNWs were contact printed on top of a SiO_2_/Si substrate and subsequently Pd contacts were prepared via an evaporation method (Figure 4a). This elegant architecture allows bi-directional sensing and, moreover, applies the contact pads as an affinity layer. Under ambient conditions an Ohmic contact is formed between Pd and Si (Figure 4b). Hydrogen adsorbs dissociatively on Pt to from hydrogens atoms (rather than H^+^ ions as the authors erroneously report), which thereafter diffuse into the Pd contacts and are believed to settle at the Pd/NW interface.

These interface charges induce a dipole layer and cause the work function of the metal to decrease effectively (Figure 4c). Eventually this leads to the formation of a Schottky barrier, which impedes holes from crossing over from the metal to the NW at the reverse biased source contact, which in turn limits the current flow.

This sensor achieved significantly higher sensitivity than (nano)sensors based on other sensing principles and enabled a reliable detection of H_2_ concentrations down to 5 ppm due to its low drift. The authors speculate that the printing process can be extended to other types of nanowire sensors either by functionalizing printed SiNWs with receptors (as in the case of DNA or protein sensing) or by heterogeneously printing different types of nanowires that are naturally selective to other gaseous or chemical agents.

**Figure 5.**
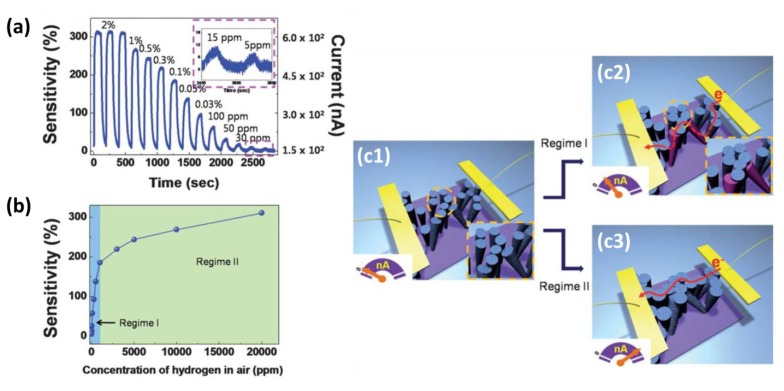
(**a**) Real-time electrical response curve of a device with vertical-standing SiNWs coated with a 7 nm thick Pd film to varying H_2_ concentrations in air at room temperature. The inset shows a clear and reversible response behavior even at very low H_2_ concentrations down to 5 ppm; (**b**) A plot of the sensitivity *vs.* H_2_ concentrations revealing two regimes with different rates of sensitivity change; (**c**) A proposed model of the hydrogen-sensing mechanism. Panel (**c1**) represents the initial devices with Pd-coated SiNWs with two on-top electrodes as indicated by the yellow lanes. The inset of the left panel shows a magnified distribution of SiNWs inside a cluster, indicating that the wires do not touch each other. Panel (**c2**) illustrates nanowire contacts inside clusters with gaps between neighboring clusters. In this case, the current flows through slanted nanowires between clusters. The inset is a magnified picture of contacted distribution of SiNWs inside a cluster. Panel (**c3**) illustrates the formation of current paths between neighboring clusters by large volume expansion of the Pd film caused by absorption of high concentrations of H_2_. This figure is composed of figures taken from reference [37], reprinted with permission from The Royal Society of Chemistry ©.

An overwhelming majority of the SiNW-based electrical devices consist of in-plane orientated SiNWs. Also, contact pads typically are prepared on each terminal side of the NW. However, Lee and co-workers reported a non-classical sensor that is made from Pd-coated, vertical-standing, rough SiNWs, which showed an excellent performance in sensing H_2_ in air [37]. Using a top-down, electroless Si etching method, the authors achieved vertical-standing SiNWs with a controllable density. The NWs were reported to be rough, although no quantification is given. Pd was then sputter-deposited only on the upper part of the SiNWs in the semi-dense configuration to avoid electrical short cuts between the two on-top electrodes, which were made using silver paste (Figure 5c1). The Pd-coated SiNWs showed good reversibility and excellent H_2_-sensing performance in terms of detection limit (∼5 ppm) and response time (<3 s). Figure 5a shows the real-time electrical responses of the sensor to varying H_2_ concentrations in air at room temperature. Figure 5b gives a sensitivity *vs.* H_2_ concentration plot, which reveals an interesting variation between the low and high H_2_ concentration ranges. The authors propose a model for this unusual finding: in the high H_2_ concentration range (regime II), the current flows through the Pd film deposited on the top region of the nanowires, as the nearest nanowires are connected by a rather large volume expansion of the Pd film after H_2_ absorption. In the low H_2_ concentration range (regime I), it is hard to make a contact between neighboring SiNWs with a relatively large distance between them. This is due to the smaller volume expansion of the Pd film, resulting in a smaller conductance increase. It is proposed that the current paths are now activated by the bridging effect of some slanted SiNWs. Thus, according to this proposed model, the slope in regime I is higher than the one in regime II based on the rationale that the current conduction is limited by gaps between SiNWs, and the slanted SiNWs act as conduction bridges.

**Figure 6.**
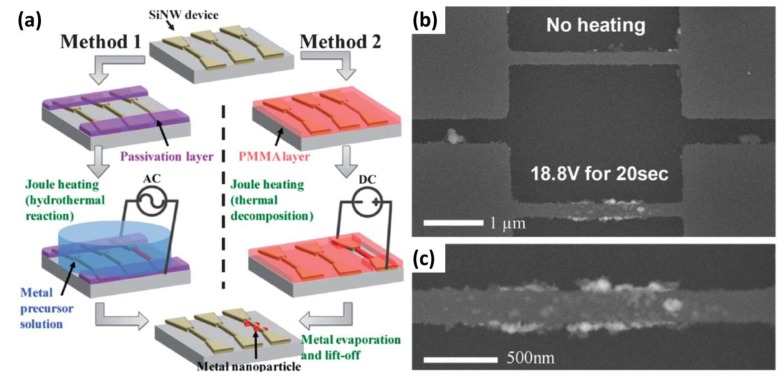
(**a**) Schematic description of surface modification by self-heating of a nanowire: in method 1, nanoparticles are formed by a hydrothermal reaction via Joule heating of a SiNW. In method 2, a metal thin film is locally deposited on a SiNW after PMMA decomposition, metal evaporation and lift-off. SEM images of the nanoparticle-decorated SiNW via Joule heating in a liquid metal precursor environment; (**b**) Pd nanoparticles selectively coated on the heated SiNW; and (**c**) high magnification SEM image of Pd nanoparticles on the heated SiNW. This figure is composed of figures taken from [40], reprinted with permission from the Royal Society of Chemistry ©.

Another study on a SiNW gas sensing application reports on the selective surface modification of the SiNWs that are part of an array [40]. The topic of selective SiNW modification was already addressed by Bunimovich *et al.* who made use of hydrosilylation chemistry to modify the surface of SiNW on a device, leaving the SiO_2_ areas in between the wires untreated [16]. Very recently, Yun *et al.* introduced a novel method for the selective surface modification of SiNWs based on nanoscale localized Joule heating [40]. In this method, Joule heating generated a localized heat along the SiNWs enabling endothermic reactions. Two different selective surface reactions were explored: hydrothermal synthesis of Pd nanoparticles and the thermal decomposition of polymer thin films to unmask one nanowire specifically before further modification (Figure 6a, methods 1 and 2, respectively). Both types of devices were exposed to 0.5% H_2_ mixed with air, showing the presence of Pd nanoparticles via measuring the nanowire resistance. Since this method does not require a tedious alignment process for selective and localized surface modification, the authors expect that integrated and multiplexed nanowire sensors can be easily developed by using their method.

### 3.2. Inorganic Vapors

#### 3.2.1. Ammonia (NH_3_)

Ammonia (NH_3_) is primarily a concern in areas of high agricultural activity, because it is a natural waste product of livestock. Industrial sources include the manufacturing of basic chemicals, metals, textiles, and paper products as well as automotive emissions. High levels of NH_3_ can result in irritation of the eyes and respiratory tracts of humans and can negatively impact wildlife, livestock, and agricultural health [72].

In 2003, Lee and co-workers demonstrated the potential of SiNW-based gas sensors in a report on the electrical response of SiNW bundles to NH_3_ and water vapors in N_2_ [41]. This work is the first example of applying SiNWs in an electrical sensor device for gas sensing purposes. It has to be noted though that in this study no single wires, but bundles of SiNWs have been used. Bundles of etched and non-etched SiNWs were made by pressing wires (∼0.4 mg) onto a surface of glass. Two electrodes were made by applying Ag glue at the two ends of the bundle. The spacing of the two electrodes was as large as 5 mm. The non-etched SiNWs hardly showed any change in electrical resistance after the adsorption of NH_3_ and H_2_O molecules, because of the presence of an amorphous silicon oxide shell on the surface of SiNWs formed during the nanowire preparation. The etched SiNWs were exposed to air, allowing the formation of native oxide. As compared to the oxide layer formed at high temperature during the SiNW growth, native oxide is less uniform and much thinner, explaining the large sensitivity difference in resistance between the etched and non-etched SiNWs.

Subsequently, several research groups have focused on improving the response to NH_3_ of sensors composed of SiNWs without any chemical functionalization. For example, Kamins *et al.* fabricated metal-catalyzed, p-doped SiNWs bridging two Si electrodes and exposed them to vapors containing NH_3_ or HCl at reduced pressure [43]. The current was measured in the dark at an applied voltage of 0.1 V. Exposure to NH_3_ resulted in a reduction of the conductance due to the adsorption of positively charged species (NH_4_^+^) on the nanowires, decreasing the density of positive mobile carriers, *i.e.*, holes in the case of p-type devices. In the case of HCl the conductance increased, which was attributed to the adsorption of Cl^−^ ions. The researchers also used additional nanowire structures as reference devices that are protected from the analyte. From the work it is not clear how this exactly was realized, but at least it is one of the few papers on SiNWs and gas sensing that address the issue of reference sensors. It is believed that one reference may serve a group of sensors, which would limit the area needed for these reference devices. Furthermore, Talin *et al.* reported a SiNW array transistor, made by a top-down technique based on nanoimprint lithography (NIL) [42]. When exposed to ammonia gas or cyclohexane solutions containing nitrobenzene or phenol, the threshold voltage of the field-effect transistor shifted. This shift was found to be proportional to the Hammett parameter (*i.e.*, a parameter related to the electron-donating or electron-withdrawing character of the substituents on the benzene ring) and the concentration of the nitrobenzene and phenol analytes. Recently, Pichon's group developed two bare SiNW-based resistors for ammonia and smoke detection, and the two types of non-intentionally doped SiNWs are fabricated by the vapor-liquid-solid (VLS) growth technique (bottom-up approach) and the so-called sidewall spacer method (top-down approach), respectively [50].

**Figure 7.**
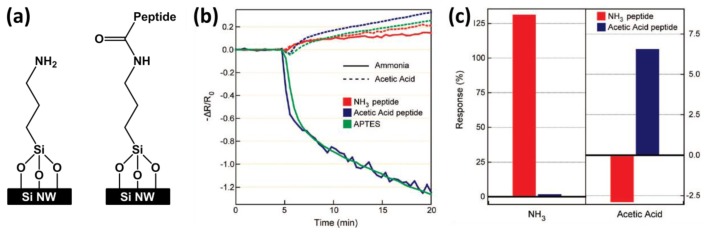
(**a**) Schematic representation of the (left) APTES- and (right) peptide-modified SiNW surface. Note that two different peptides have been used, *i.e.*, an ammonia recognition peptide and an acetic acid recognition peptide; (**b**) Electrical responses of the three different devices (APTES, NH_3_ peptide and acetic acid peptide) to ammonia and acetic acid vapors (100 ppm in N_2_) introduced to the sensing chamber after 5 min of flowing N_2_; (**c**) Conductance responses of the peptide-nanowire hybrid sensors, averaged over a 5 min time window of target vapor exposure (starting 10 min after the analyte gas exposure), and normalized to the amine-terminated sensor. Figures b,c are taken from [44], reprinted with permission from American Chemical Society ©.

Based on the well-established “lock-and-key” interactions to achieve high selectivity, particularly used in biosensors, Heath's group developed novel SiNW-based sensors modified by peptides for the selective detection of ammonia and acetic acid vapors [44]. First, the SiNWs were fabricated by the SNAP method and treated with O_2_ plasma, followed by the immersion in the surface modifying reagent 3-aminopropyltrimethoxysilane (APTES) solution to realize amine-terminated SiNW surfaces (Figure 7a, left). Next, oligopeptides with the desired recognition sequences (NH_3_ and CH_3_COOH) were synthesized, and coupled to the APTES-modified SiNWs (Figure 7a, right). Upon exposure to NH_3_ and acetic acid vapors, the hybrid materials demonstrated the ability of discriminating the target molecules at low concentrations from, what the authors call “chemically camouflaged” mixtures. The electrical responses are given in Figures 7b,c. It was concluded that the results serve as a model platform for what can be achieved in terms of selective and sensitive “electronic noses”.

**Figure 8.**
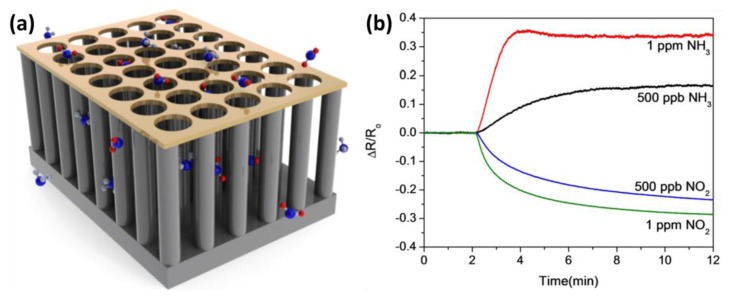
(**a**) Schematic illustration of the periodically porous top electrode (PTE) nanowire array sensor concept; (**b**) Sensor response to various concentrations of NO_2_ and NH_3_ following 2 min of clean air: 1 ppm of NH_3_ (red), 500 ppb of NH_3_ (black), 1 ppm of NO_2_ (green) and 500 ppb of NO_2_ (blue) at ∼30% RH. This figure is composed of figures taken from [48], reprinted with permission from IOP Publishing Ltd ©.

To realize high sensitivity in gas sensing, In *et al.* reported a novel method to fabricate ordered arrays of vertically aligned SiNWs provided with a periodically porous top electrode [48]. Two separate nanosphere lithography steps were used to fabricate large, well-ordered arrays of vertical nanowires. Subsequently, a periodically porous top electrode (PTE) was prepared, making consistent electrical connections to every single nanowire in the array (Figure 8a). With this sensor configuration a fast and highly sensitive detection of NH_3_ and NO_2_ in humidified air has been shown (Figure 8b). NO_2_ detection down to 10 ppb has been demonstrated and the sensor response times were found to be in the range of 2 to 8 min. The authors attributed the high sensitivity to the vertical array configuration, enabling the deposition of top electrode material, while the pores in this top electrode significantly improve the sensing response times as analyte gases can pass through easily.

In 2012, Ni and co-workers developed two types of resistor-based sensors by growing (bare) SiNWs from gold-modified, interdigitated, comb-shaped and V-shaped groove structures [51]. The resulting devices were tested as a chemical sensor by exposing them to different ammonia concentrations in nitrogen. The V-shaped groove structures provide the possibility to be assembled with a capping polydimethylsiloxane (PDMS) mold, and is also more compatible with planar technologies. A recent study shows that the modification of H-terminated SiNWs with tellurium nanoparticles (Te NPs) by the reduction of Na_2_TeO_3_ [53]. These devices were sensitive to ammonia and propylamine, which was rationalized as following: ammonia and propylamine reduce the tellurium oxide (present on the surface of Te NPs), decreasing the majority carrier density in the Te, which in turn decreases the conductivity of the sensor.

#### 3.2.2. Humidity (H_2_O)

Sensing humidity is an important application for chemical sensors and is extensively used in our daily life. Humidity sensors can monitor the environmental moisture for human comfort and can also be used in automotive, medical, construction, semiconductor, meteorological, and food processing industries [73]. As a semiconductor material, SiNWs can be used for humidity sensing, since it is sensitive to environmental humidity. Jie *et al.* showed that n-type SiNWs exhibit p-type characteristics when exposed to air [74]. The authors explained this by the presence of water molecules that adsorb on the NWs due to the large number of dangling bonds and surface defects. Also the effect of doping on the SiNW sensitivity to humidity has been reported [52]. In 2009, Zhang and co-workers developed a capacitive humidity sensor based on the capacitance variations due to the adsorption/desorption of water vapor on SiNWs [45]. In a subsequent study, the SiNWs were made hydrophobic by the modification with hexamethyldisilazane (HMDS) [49]. While the sensitivity of these layers was lower as compared to oxide-covered SiNW, the hysteresis, linearity and response time remarkably improved. Hsueh *et al.* reported SiNW-based humidity sensors by growing SiNWs directly on a glass substrate by a low-temperature VLS process without intentional doping [47]. The deposition of a gold layer on the top of the standing NWs made their behavior p-typed. The current decreased monotonically upon increasing the relative humidity from 30% to 95%. These results suggest that SiNWs prepared on glass substrate are potential useful as a cost-effective alternative for humidity sensing. Meanwhile, Passi *et al.* fabricated single-crystalline SiNWs using the top-down approach, and studied the transfer characteristics and sensing properties of the SiNWs with and without surface functionalization under ambient conditions [46]. To reduce the adsorption of H_2_O molecules onto the surface of SiNWs, surface modification was performed by grafting either 3-(4-ethynylbenzyl)-1,5,7-trimethyl-3-azabicyclo[3.3.1] nonane-7-methanol (EBTAM, a receptor molecule for a nerve agent, compound **1** in Figure 10) on H-terminated Si surfaces, or octadecyltrichlorosilane (OTS) onto native oxide formed on the SiNW surface. A reduction of hysteresis in the *I*–*V* curves is observed, from which the authors conclude that surface functionalization is important to avoid any undesired environmental effect on the transport properties of the SiNWs.

### 3.3. Organic Compounds

#### 3.3.1. Explosives

**Figure 9.**
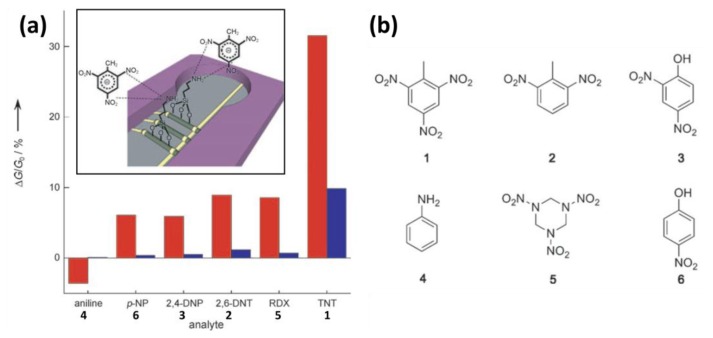
(**a**) The response of an APTES-functionalized SiNW device to (red) 5 μM solutions and (blue) 5 nM solutions. The inset shows the two different kind of interactions between TNT and the NH_2_-terminated SiNWs; (**b**) Molecular structures of six different N-containing compounds: (**1**) TNT; (**2**) 2,6-dinitrotoluene; (**3**) 2,4-dinitrophenol; (**4**) aniline; (**5**) (1,3,5-trinitroperhydro-1,3,5-triazine; and (**6**) p-nitrophenol. Figure based on graphs taken from [55] and used with permission from Wiley-VCH©.

With the increased threat of terrorist activities, there is an unfortunate need for the detection of chemical vapors indicative of malicious intent. One of the most commonly used high explosives in the past years is 2,4,6-trinitrotoluene (TNT). TNT does not only poses a security threat, but it is also of great environmental concern because of soil and water contamination. However, the low volatility of most explosives makes it challenging to develop and integrate methods to detection traces of explosives. One of the lowest detection limits for TNT (as low as 10^−2^ ppt in air) was realized by the Patolsky group using large-scale arrays of SiNW-FET devices. The devices were chemically modified with APTES to obtain an amine-terminated layer [55]. The thickness of the APTES layer was found to be ∼6.5 to 12 Å, *i.e.*, between a monolayer and a bilayer. The authors note that the structure and thickness of APTES films indeed are governed by the deposition time and the composition of the silane solution [75]. No attempts to prepare a true APTES monolayer were reported in this study. The authors continue their work by describing two kinds of strong (reversibly) interactions may occur between the electron-deficient aromatic ring of TNT and the electron-rich amino group of APTES (Figure 9a, inset). First, the charge transfer from amino groups to aromatic rings leads to the formation of so-called Meisenheimer complexes. Second, a TNT molecule is a Brønsted-Lowry acid, which can be deprotonated at the methyl group by a basic amine. In both cases, charges are formed near the sensing surface, thus leading to abrupt changes in the conductance of the device. The cross-reactivity of the devices was studied using structurally related compounds (Figure 9b). The results clearly show a strong preference for binding TNT over other similar compounds (Figure 9a). It should be noted that all these experiments were performed using 0.1% DMSO/H_2_O solutions of the compounds. In a final experiment it is shown that TNT can also be detected directly in air at low concentrations of TNT (between ppb and ppt concentrations), with unprecedented sensitivities down to at least 10^−2^ ppt in air.

More recently, Wang and co-workers reported a chemiresistive device based on arrays of hydrogen and oxygen plasma-treated SiNWs for the detection of the vapors of common nitro explosives and their degradation by-products [56]. The following compounds were tested: 2,4-dinitrotoluene (DNT), 2,4,6-trinitrotoluene (TNT), cyclotrimethylenetrinitramine (RDX), pentaerythritoltetranitrate (PETN), 2,4,6-trinitrophenol (picric acid) and an explosive degradation by-product, 2-nitrotoluene. In their work, the width of nanowires was varied (100, 200 and 400 nm). The sensitivity—defined as the relative resistance change due to the presence of chemicals—was found to increase for decreasing cross-sections of the nanowires. Both plasma treatments can significantly improve the sensitivity and response times. This was explained by its cleaning effect providing more adsorption/binding sites for the target molecules. It was further rationalized by the authors that the oxygen-plasma prepared surface Si-OH groups might form charge transfer complexes with the nitro groups of nitro-containing explosives to strengthen the chemiresistive response. Surface studies such as X-ray photoelectron spectroscopy (XPS) and Hall measurements at room temperature were performed on reference Si samples to confirm that oxygen plasma treatment changes the type of majority carriers from p to n and inverts the sign of the resistance change.

#### 3.3.2. Nerve Agents

**Figure 10.**
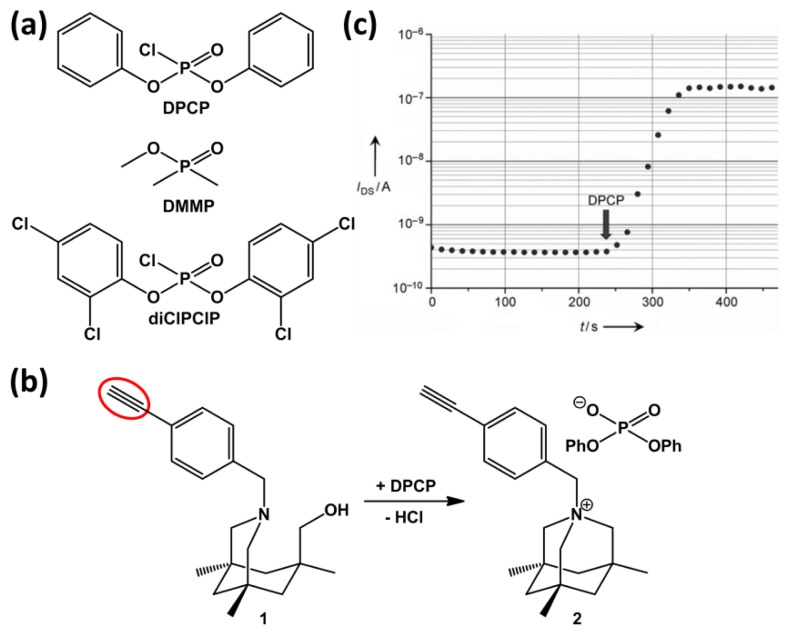
(**a**) Molecular structure of DPCP and two structurally related phosphates; (**b**) Sensitive receptor towards DPCP. Compound **1** converts into compound **2** upon exposure nerve agent simulant DPCP. The red oval highlights an alkyne group, which can react with H-terminated Si to form a stable Si-C bond; (**c**) Change in drain current of a SiNW FET device that has been modified with compound **1** upon exposure to DPCP, which was introduced at *t* = 240 s. Figure c is taken from [54] with permission Wiley-VCH.

The ability to detect minute traces of chemical warfare agents (CWAs) is mandatory both for military forces and homeland security, since CWAs can be fatal even at low concentration levels. Towards the development of sensors for CWAs, specifically nerve agents, simulants are often used, such as dimethyl methylphosphonate (DMMP) and diphenylchlorophosphate (DPCP). Several groups have made efforts towards developing SiNWs-based sensors for nerve agents. In a very brief conference proceeding without any sensing data, Lee and co-workers report on the electrical detection of the vapor of DMMP in N_2_ using a device with bare SiNWs in a resistor mode [57]. More interestingly, Simonato and Raskin and co-workers reported a highly sensitive detection of DPCP (Figure 10a, top) using chemically functionalized silicon nanoribbon FETs [54] and silicon nanowire FETs [59,58], respectively. In their work, the silicon nanostructures were fabricated and then functionalized by covalent grafting through thermal hydrosilylation of compound **1** onto the HF pre-treated substrates. This type of chemistry results in an organic monolayer that is linked via a stable Si-C bond. Compound **1** is known to react cleanly and irreversibly with DPCP to produce the so-called aza adamantane quaternary ammonium salt, compound **2** (Figure 10b).

Figure 10c shows the change in *I*_DS_ when a modified device is exposed to 500–800 ppb of DPCP in air. With the exception of bis-dichlorophenylchlorophosphate (diClPClP), the use of more than about 20 other VOCs, including DMMP, showed hardly any change in the drain current (see Figure 10a for the structures of diClPClP and DMMP). The absence of response in the case of DMMP was explained by the low chemical reactivity of DMMP with compound **1** due to the electrodonating properties of the methoxy group [59]. In our opinion, another explanation may be related to a reaction between the P–Cl functionality and the primary alcohol of compound **1** to form an intermediate. This would also explain the absence of response for DMMP as a methoxy group is less reactive than Cl. Also, it would explain the sensor response of diClPClP as it contains a P–Cl functionality, just like DPCP. The authors realize that apart from the positively charged nitrogen present in compound **2** also the negatively charged counter ion is present, making the monolayer likely to remain neutral. It was further rationalized that the drain current change upon DPCP exposure could not be explained by changes in the density of the Si surface states. This made them conclude that the drain current changes could be explained by a charge transfer between Si and the molecules, which in turn leads to interface dipoles and a change in the Si surface potential. 3D technology-computer-aided-design (TCAD) simulations before and after gas detection have been performed to gain insight into the physical mechanisms involved in the gas detection and to investigate the impact of the surface-to-volume ratio on sensor sensitivity [58]. It was found that by reducing the nanowire width from 1 μm to 25 nm, the sensitivity only slightly improved, while the *V*_bg_ window, in which the high sensitivity is reached, significantly enlarged.

#### 3.3.3. Volatile Organic Compounds (VOCs)

Volatile organic compounds (VOCs) are carbon-based chemicals that have a relatively high vapor pressure at room temperature, which is the result from their low boiling points. Most odors consist of VOCs. Other examples include biogenic VOCs produced by plants, which are involved in plant growth, development, reproduction and defense. However, some VOCs are dangerous to human health or cause harm to the environment. Moreover, VOC biomarkers that can be present in the exhaled breath of humans could be used for the detection of some diseases, including lung cancer [76]. Thus, the ability to monitor VOCs is important for environmental safety and medical reasons.

In 2011 Niskanen *et al.* reported SiNWs that can be used as versatile chemical vapor sensors without elaborate functionalization [60]. Obviously, functionalization of the SiNW surface can help the recognition task greatly, as also becomes clear from other examples in the current review. Nonetheless, their research is a proof-of-principle demonstration that even non-functionalized nanowires can distinguish between various VOCs in—what the authors call—completely uncontrolled ambient conditions with the help of elementary machine learning. The results show that they are able to distinguish between acetone, ethanol and water with 100% accuracy, while methanol, ethanol and 2-propanol were classified with 96% accuracy in ambient conditions. The mechanism is not discussed in great detail, although they conclude it is clear that the sensing is based on the field effect and that the dielectric coupling effect is likely to be dominant owing to large dielectric constants of the studied compounds (ranging from 18 to 80). In fact, the authors emphasize that the strength of their classification technique means that knowledge of the exact nature of the sensing mechanism is not required. The set of responses was found to be unpredictable, making them conclude that this approach would be unsuitable for traditional sensing based on deterministic methods. On the other hand, the identification and extrapolation of sensing patterns via supervised machine learning seems achievable.

**Figure 11.**
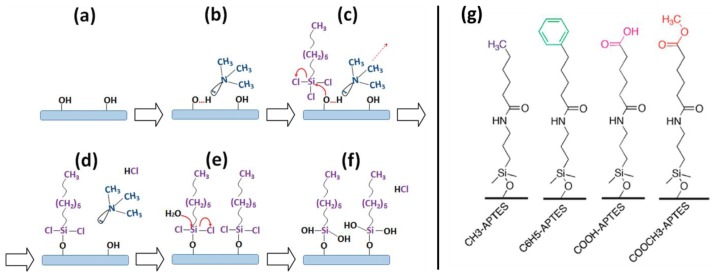
Simplified scheme of the attachment of hexyltrichlorosilane (HTS) to the SiO_2_ surface: (**a**) Preparation of surface hydroxyl (Si–OH) groups; (**b**) Exposure to trimethylamine (TMA) to form a hydrogen bond with the Si–OH group to make the oxygen atom more nucleophilic; (**c**) Exposure to 1.5 mM of HTS in chloroform. At this stage, the oxygen atom of the Si–OH group attacks the silicon atom of HTS to form a Si–O–Si bond as given in (**d**); (**e**) The presence of water residues assists the replacement of chlorine atoms by OH groups to form the final product given in (**f**); (**g**) Schematic illustration of molecular layer with different functional groups on the SiNW surface. Figure a–f and Figure g are taken from [63] and [64], respectively, reprinted with permission from American Chemical Society ©.

Over the past few years the research group of Haick published a number of interesting and systematic studies on the fundamentals and applications of functionalized SiNW-based FETs for VOC detection [61–66]. Both polar (water, ethanol, 1-butanol, 1-hexanol, 1-octanol and 1-decanol) and nonpolar (*n*-hexane, *n*-octane and *n*-decane) VOCs have been studied in oil-free air having 15% relative humidity. It was shown that the formation of silane monolayers having a low fraction of Si–O–Si bonds between the adjacent molecules (*i.e.*, no cross linking) greatly enhanced the sensitivity toward nonpolar VOCs [61]. In more detail, a monolayer of hexyltrichlorosilane (HTS) was prepared using a two-step, amine-promoted reaction procedure (Figure 11a–f). The improved sensitivity was attributed to the adsorption of nonpolar VOCs between or on top of the alkyl chains and/or in the pinholes of the monolayer, inducing conformational changes in the organic monolayer. This affects the dielectric constant, the effective dipole moment of the organic monolayer, and/or the charged surface state density of the SiO_2_/monolayer interface. These effects in turn change the conductivity of the SiNW.

In a second study the detection of nonpolar species with HTS-modified devices is further studied experimentally, while also the detection process has been modeled based on changes in the carrier mobility, voltage threshold, off-current, off-voltage, and subthreshold swing of the devices [62]. The detection of the nonpolar species was attributed to molecular gating and based on two indirect effects: (i) a change in the dielectric medium close to the SiNW surface and (ii) a change in the charged surface states at the functionality of the SiNW surface. Subsequently, the devices were modified with alkyl trichlorosilanes with different alkyl length to study the interactive effect of hysteresis and surface chemistry [63]. The density of the exposed or unpassivated Si–OH groups (trap states) on the SiNW surface was found to play by far a crucial effect on the hysteresis characteristics of the gated silicon nanowire sensors, relative to the effect of hydrophobicity or molecular density of the organic monolayer.

APTES-modified devices were also functionalized with acyl chlorides (with different chain length) to form amide bonds [65]. The resulting devices showed a negative response in the threshold voltage changes and the relative hole mobility changed upon exposure to polar and to nonpolar VOCs. In reference experiments, the bare SiNW FET showed no clear response in the threshold voltage to VOCs. For each VOC, the change in threshold voltage increased with the chain length of the molecular modification, while the changes in hole mobility were found to be constant. Data analysis with an electrostatic-based model suggests that the sensor response in terms of the change in threshold voltage depends on the concentration and the vapor pressure of the VOC as well as on the VOC–molecular layer binding energy, and VOC adsorption-induced dipole moment changes of the molecular layer. Also 4 different acyl chlorides were used in the formation of amides, resulting in layers with different terminal functionalities (Figure 11g) [64]. It was concluded that the electron-donating/withdrawing properties of the functional groups in these layers likely control the dipole moment orientation of the adsorbed VOCs and, consequently, determine the direction of the change in threshold voltage. Additionally, it was found that the diffusion of VOCs into the molecular layer likely determined by the type of functional groups, is the main reason for change in the hole mobility. In their most recent paper, a systematic, comparative analysis was performed on SiNW sensors functionalized with a series of silanes having different electron donating/withdrawing end groups and different chain lengths [66]. None of the separate sensors showed sufficient chemical and quantitative selectivity to realize accurate VOC detection. However, using discriminant factor analysis (DFA), the performance of the molecularly modified SiNW FETs made it possible to discriminate between polar and nonpolar VOCs, as well as between the separate VOCs inside each group.

## 4. Conclusions and Outlook

SiNW-based sensors have demonstrated sensitivity to a wide variety of gas-phase analytes that are important in industrial, environmental, and personal safety and in medical and even military scenarios, as summarized in Table 1. Close to half of these studies are on top-down prepared nanowires. While top-down approaches are generally believed to be beneficial in terms of sensitivity and reproducibility due to better contacts and higher uniformity, the large variety of approaches as listed in Table 1 makes it difficult to say whether this is also the case for the studied nanowires reviewed here. Interestingly, devices prepared from the bottom-up and top-down approaches have been used in two studies [50,60]. However, differences in the oxide layer thickness or Si crystallinity and differences in the level of impurities prevent a clear, direct comparison, as recognized by the authors. In any case, top-down approaches are preferable in the preparation of devices that consist of large arrays of SiNWs to keep the difference between the nanowires and their contacts (and hence their performance) as low as possible. Also, it should be realized that the bottom-up nanowires are typically wires indeed, while in the case of top-down nanowires the cross section of the wire is not spherical, but rectangular, trapezoidal or triangular. In essence, for “nanowires” with a rectangular cross section having a rather high width-to-height aspect ratio, nanosheets or nanoribbons would be a more appropriate name.

About one third of the papers on gas phase sensors/detectors that consist of SiNWs make use of the bare NW surface, *i.e.*, oxide surface. The other papers show that surface modification can be advantageous in terms of selectivity, e.g., Pt for H_2_ sensing as shown in different studies or the use of recognition peptide sequences for NH_3_ and CH_3_COOH [44]. Surface modification can also be useful for mechanistic studies as illustrated by the preparation of organic monolayers with different degrees of cross linking [61] and chain length [63,65]. The development of new and the implementation of existing functionalization strategies will enlarge the number of selective (gas phase) sensors. So far, two approaches have been reported to modify individual nanowires present in an array, one based on the tuning of etching conditions [16] and the other one on nanoscale localized Joule heating [40]. Wider implementation of these methods and the development of new strategies in this regard will further stimulate multiplexing sensor applications. We anticipate that also recent work on the identification of sensing (recognition) patterns via advanced analytical procedures based on data obtained with SiNW-based devices [44,60,66] will contribute to further developments in the analysis of complex biological and chemical gas mixtures.

Apart from sensitivity and selectivity, also the speed of response and stability are two important issues in the field of sensor development in general. Response times of the reviewed studies are typically at the time scale of seconds to min. However, faster response times are needed for some applications, e.g., an automotive exhaust sensor that requires a response time of 10 ms in order to enable feedback control [77]. Furthermore, as the degradation of the selector layer will reduce sensor the performance, stability is a key issue to future applications that require operation under harsh temperature and environmental conditions. While response reversibility has been shown in most, if not all cases, stability studies over time have not been addressed extensively. This will be a huge challenge for organic selector layers in particular.

To conclude, as investigations of the influence of Si nanowire dimensions on sensor sensitivities are just beginning to be reported, more work in this direction is needed. Clearly, a lot can be learned of the more extensive work on the application of SiNW-based devices in the liquid environment. Improvements of the four S's—sensitivity, selectivity, speed and stability—are essential in any discussion of chemical sensor development [77], but it must be realized that some relevant topics can be studied within in the domain of gas phase sensors only. Obviously this holds for the development of suitable gas selector layers, but also for a rather fundamental topic on the effect of having no possibility of using a (typical) liquid-gate reference electrode on the sensor performance. In general, the results presented in this review confirm the applicability of sensors that consist of SiNWs for a wide range of gas sensing applications. It is anticipated that this scientific and technological work soon may result in spin-off commercial products that will a have substantial contribution to the quality of life.

## Figures and Tables

**Table 1.** Overview of SiNW-based sensors for the detection of inorganic gases and vapors and organic explosives, nerve agents and VOCs.

		**Year**	**First Author**	**Approach ^a^**	**SiNW Size ^b^**	**Functionalization**	**Principle**	**Target(s)**	**Sensitivity/ Measured Concentration ^c^**	**Ref.**
**Inorganic Compounds**	**Gases**	2007	McAlpine	TD (p)	18 nm (w) 5 μm (l)	Bare	FET	NO_2_	20 ppb (s)	[31]
		2007	Chen	BU (n) *	80–200 nm (d) ∼2.5 μm (l)	Pd NPs	FET	H_2_	5% (mc)	[32]
		2009	Wan	TD (p)	75, 130 nm (w) 20 μm (l)	Bare	Resistor	NO_2_, NH_3_	250 ppm (mc) 250 ppm (mc)	[33]
		2009	Peng	TD (n)	No data	Bare	Resistor	NO	500 ppb (mc)	[34]
		2010	Gao	TD (p)	22, 75 nm (w) 2 μm (l)	Bare	Resistor	NO_2_	250 ppm (mc)	[35]
		2010	Skucha	BU (p)	30 nm (d) 2 μm (g)	Bare	FET	H_2_	3 ppm - 5% (mc)	[36]
		2011	Noh	TD (n)	30–40 nm (d) 20 μm (h)	Pd coating	Resistor	H_2_	∼5 ppm (s)	[37]
		2011	Gao	TD (p)	22–100 nm (w) 2 μm (l)	Bare	Resistor	NO_2_	250 ppm (mc)	[38]
		2012	Cuscunà	BU (p) ^&^	150–200 nm (d) 5–8 μm (l)	Amino groups (via ammonia plasma)	Resistor (IDE) ^$^	NO_2_	10 ppb (mc)	[39]
		2013	Yun	TD (p)	100 nm (w) 3 μm(l)	Pd NPs	Resistor	H_2_	0.5% (mc)	[40]
	**Vapors**	2003	Zhou	BU (n) ^&^	∼20 nm (d) 5 mm (g)	Si-H (via HF etch)	Resistor	NH_3_, H_2_O	1,000 ppm (mc), 60% humidity (mc)	[41]
		2006	Talin	TD (p)	76 ± 5 nm (d) 4 μm (g)	Bare	FET	NH_3_	No data	[42]
		2006	Kamins	BU (p)	∼200 nm (d) 7 μm (l)	Bare	Resistor	NH_3_, HCl	Sat. con. (mc) ^8^	[43]
		2008	McAlpine	TD (p)	16 nm (w) 5 μm (l)	Peptides	Resistor	NH_3_, AcOH	100 ppm (mc) 100 ppm (mc)	[44]
		2009	Li	TD (n)	80 μm (l)	Bare	Resistor	Humidity	10%–100% (mc)	[45]
		2011	Passi	TD (p)	25 nm (w) 300 nm (l)	DPCP receptor ^1^ OTS ^2^	FET	Humidity	Ambient (mc)	[46]
		2011	Hsueh	BU (p) ^&^	90 nm (d) 1.6 μm (l)	Au	Resistor	Humidity	30%–95% (mc)	[47]
		2011	In	TD (p)	∼200 nm (d) 4–6 μm (l)	PTE from metal ^3^	Resistor	NH_3_, NO_2_	1 ppb (s) 10 ppb (s)	[48]
		2011	Chen	TD (n)	55 μm (l)	Hexamethyldisilazane	Resistor (IDE) ^$^	Humidity	11.3%–93% (mc)	[49]
		2012	Demami	BU (n)	100 nm (d) 20 μm (l)	Bare	Resistor	NH_3_, smoke	No data	[50]
				TD (n)	100 nm (d) 10 μm (l)					
		2012	Ni	BU (n)	150 nm (d) >20 μm (l)	Bare	Resistor	NH_3_	175–700 ppm (mc)	[51]
		2013	Taghinejad	BU (n)	20–600 nm (d)	Bare	Resistor	Humidity	42%–92% (mc)	[52]
		2013	Yang	BU ^#^	50 nm (d)	Te NPs	Resistor	NH_3_, propylamine	10–400 ppm (mc) 5–25 ppm (mc)	[53]
**Organic Compounds**	**Explosives**	2010	Clavaguera	TD (p)	0.2, 1, 4 μm (w) 2, 4 μm (l)	DPCP receptor ^1^	FET	DPCP ^1^	500–800 ppb (mc)	[54]
		2010	Engel	BU (p)	2 μm (g)	APTES ^4^	FET	TNT ^6^	10^-2^ ppt (s)	[55]
		2012	Wang	TD (p)	100, 200, 400 nm (w) 100 μm (l)	H_2_/O_2_ plasma treatment	Resistor	Nitro explosives	Sat. con. (mc) ^8^	[56]
	**Nerve Agents**	2010	Kim	BU (p)	50 nm (w) 3 μm (l)	Bare	Resistor	DMMP ^7^	No data	[57]
		2011	Passi	TD (p)	25 nm (w) 300 nm (l)	DPCP receptor ^1^	FET	DPCP ^1^	500-800 ppb (mc)	[58]
		2011	Clavaguera	TD (p)	0.2, 1, 4 μm (w) 2, 4 μm (l)	DPCP receptor ^1^	FET	DPCP ^1^	500 ppb (mc)	[59]
	**VOCs**	2011	Niskanen	BU ^#^	30–70 nm (d) ≥400 nm (l)	Bare	FET	Different VOCs	No data	[60]
				TD ^#^	50 nm (d)					
		2011	Paska	BU (p)	60 nm (d) 2 μm (g)	CH_3_(CH_2_)_15_SiCl_3_	FET	(Non) polar VOCs	ppm level (mc)	[61]
		2012	Paska	BU (p)	50 ± 5 nm (d) 2 μm (g)	CH_3_(CH_2_)_15_SiCl_3_	FET	Nonpolar VOCs	ppm level (mc)	[62]
		2012	Paska	BU (p)	50 ± 5 nm (d) 2 μm (g)	CH_3_(CH_2_)_n_SiCl_3_ (n + 1 = 3, 6, 8, 12, 18)	FET	(Non) polar VOCs	ppm level (mc)	[63]
		2013	Wang	BU (p)	40 ± 8 nm (d) 2 μm (g)	APTES + 4 different carbonyl chlorides ^5^	FET	(Non) polar VOCs	0.01–0.08 p_a_/p_o_ (mc) ^9^	[64]
		2013	Wang	BU (p)	40 ± 8 nm (d) 2 μm (g)	APTES + CH_3_(CH_2_)_n_COCl (n + 1 = 6, 7, 10, 11) ^5^	FET	(Non) polar VOCs	0.01–0.08 p_a_/p_o_ (mc) ^9^	[65]
		2013	Ermanok	BU (p)	40 ± 8 nm (d) 2 μm (g)	Silanes with different chain lengths and end groups	FET	(Non) polar VOCs	0.01–0.08 p_a_/p_o_ (mc) ^9^	[66]

a TD: top down; BU: bottom up; p: positive type doping; n: negative type doping;

b w: width; l: length; d: diameter; g: gap between electrode contacts;

c s: sensitivity; mc: measurement concentration;

* no doping, but n-type behavior;

& type of doping not reported, but deduced from the sensor response data;

# non-doped;

$ interdigitated electrode;

1 DPCP: diphenylchlorophosphate (Figure 10a); its receptor is 3-(4-ethynylbenzyl)-1,5,7-trimethyl-3-aza-bicyclo[3.3.1]nonane-7-methanol (EBTAM or TABINOL, compound **1** in Figure 10b);

2 OTS: octadecyltrichlorosilane;

3 PTE: porous top electrode (Figure 8a);

4 APTES: 3-aminopropyltriethoxysilane (Figure 7a);

5 APTES-modified SiNW were reacted with acyl chlorides to form amides (Figure 11g);

6 TNT: 2,4,6-trinitrotoluene;

7 DMMP: dimethylmethylphosphonate (Figure 10a);

8 Sat. con.: saturated concentration;

9 *p*_a_: VOC's partial pressure, *p*_o_: vapor pressure.
